# TMAO Aggregates Neurological Damage Following Ischemic Stroke by Promoting Reactive Astrocytosis and Glial Scar Formation *via* the Smurf2/ALK5 Axis

**DOI:** 10.3389/fncel.2021.569424

**Published:** 2021-03-18

**Authors:** Haibo Su, Shaoping Fan, Lingqiong Zhang, Hui Qi

**Affiliations:** ^1^Department of Neurosurgery, Peking University Shenzhen Hospital, Shenzhen, China; ^2^Department of Neurosurgery, The People's Hospital of Longhua District, Shenzhen, China; ^3^Department of Hand Surgery, Peking University Shenzhen Hospital, Shenzhen, China

**Keywords:** ischemic stroke, trimethylamine N-oxide, SMAD specific E3 ubiquitin-protein ligase 2, activin receptor-like kinase-5, glial scarring, neurological function

## Abstract

Ischemic stroke has been reported to cause significant changes to memory, thinking, and behavior. Intriguingly, recently reported studies have indicated the association of Trimethylamine N-oxide (TMAO) with the acute phase of ischemic stroke. However, the comprehensive underlying mechanism remained unknown. The objective of the present study was to investigate the association between TMAO and recovery of neurological function after ischemic stroke. For this purpose, a middle cerebral artery occlusion/reperfusion (MCAO/R) rat model was established and treated with TMAO or/and sh-ALK5, followed by the neurological function evaluation. Behaviors of rats were observed through staircase and cylinder tests. Moreover, the expression of Smurf2 and ALK5 was detected by immunohistochemistry while expression of GFAP, Neurocan, and Phosphacan in brain tissues was determined by immunofluorescence. Thereafter, gain- and loss-of-function assays in astrocytes, the proliferation, viability, and migration were evaluated by the EdU, CCK-8, and Transwell assays. Besides, Smurf2 mRNA expression was determined by the RT-qPCR, whereas, Smurf2, ALK5, GFAP, Neurocan, and Phosphacan expression was evaluated by the Western blotting. Finally, the interaction of Smurf2 with ALK5 and ALK5 ubiquitination was assessed by the co-immunoprecipitation. Notably, our results showed that TMAO promoted the proliferation of reactive astrocyte and formation of glial scar in MCAO/R rats. However, this effect was abolished by the Smurf2 overexpression or ALK5 silencing. We further found that TMAO upregulated the ALK5 expression by inhibiting the ubiquitination role of Smurf2. Overexpression of ALK5 reversed the inhibitory effect of Smurf2 on astrocyte proliferation, migration, and viability. Collectively, our work identifies the evolutionarily TMAO/Smurf2/ALK5 signaling as a major genetic factor in the control of reactive astrocyte proliferation and glial scar formation in ischemic stroke, thus laying a theoretical foundation for the identification of ischemic stroke.

## Introduction

Stroke is considered a leading cause of disability and death across the globe whilst ischemic stroke is a refractory brain injury resulting in substantial loss of function (Roy-O'Reilly and McCullough, 2018; Ozaki et al., 2019). As a heterogeneous syndrome, stroke can be induced by various modifiable and non-modifiable risk factors, including age, gender, hypertension, diet, and inflammatory disorders (Boehme et al., 2017). Of note, the onset and clinical outcomes of ischemic stroke have been reported to be closely correlated with age; therefore, the effects of currently available treatment modalities are limited in elderly patients (Chen et al., 2010). However, for young adults aged between 18 and 50 years, optimal management has been indicated to be distinctive from elder patients, which requires further exploration. On the other hand, long-term care costs are also very high due to their long-life expectancy (Ekker et al., 2018). Therefore, effective therapeutic strategies are prerequisite for the improvement of prognosis.

Recently, trimethylamine N-oxide (TMAO), a metabolite released from phosphatidylcholine and choline depending on gut microbiota, has emerged as a key regulator in cardiovascular diseases from perspectives of diagnosis, prevention, and management (Kanitsoraphan et al., 2018). In the context of ischemic stroke, a significantly high level of TMAO has been identified as an independent predictor (Liang et al., 2019; Nam, 2019). Notably, TMAO has also been reported to reduce the expression of Smad ubiquitination-related factor 2 (Smurf2) in cardiac fibrosis after myocardial infarction (Yang et al., 2019). Besides, the implication of Smurf2 in ischemic stroke has recognized its pivotal role in mediating neuronal differentiation and functional recovery (Yu et al., 2013). Intriguingly, the formation of glial scar and extensive glial response to brain injuries have also been detected in animal models of stroke, which are widely used to mimic clinical conditions of a human stroke to investigate possible therapeutic targets (Huang et al., 2014; Hermann et al., 2019). Furthermore, activing-like kinase 5 (ALK5), a receptor for transforming growth factor β1 (TGFβ1), has been deciphered to be functional for reactive astrogliosis as well as glial scar formation in stroke to exert an inhibitory effect on the restoration of neurological function (Zhang et al., 2018). More importantly, Smurf2 (a member of the HECT family of E3 ubiquitin ligases) has been demonstrated to regulate the degradation of TGFβ receptor-I in a negative manner (Chae et al., 2019). The present study speculated the regulatory role of Smurf2 in the degradation of ALK5 in ischemic stroke. Hence, given the aforementioned reports, we proposed a bold hypothesis that TMAO might be involved in the modulation of ischemic stroke through interaction with Smurf2 and ALK5 to modulate reactive astrogliosis and glial scar formation. Thus, the present study was conducted to provide a novel insight into the development of effective treatment modes for patients with ischemic stroke.

## Materials and Methods

### Ethics Statement

The experiment procedures involving animals were approved by the Animal Ethics Committee of Peking University Shenzhen Hospital. All animals received humane care according to the *Guide for the Care and Use of Laboratory Animals* published by the US National Institutes of Health. Extensive efforts were made to ensure minimal suffering of the included animals.

### Establishment of Middle Cerebral Artery Occlusion/Reperfusion (MCAO/R)-Induced Rat Models

Ninety adult male Sprague Dawley (SD) rats (weighing 200–250 g) were purchased from the Medical Experimental Animal Center of Guangdong Province (Guangdong, China). Rats were housed in a standard environment of 22°C and 70% humidity with a 12-h light/dark cycle and free access to water and food. Afterward, rats were fed with the drinking water dissolved with TMAO (120 mg/kg; 317594, Sigma-Aldrich, St. Louis, MO, USA) for consecutive 3 weeks before MCAO/R modeling (Meng et al., 2019). Two days before MCAO/R, rats were anesthetized by intraperitoneal injection of 1% sodium pentobarbital at a dose of 30 mg/kg and placed on a stereotaxic device (RWD Life Science, San Diego, CA, USA). The 5 μL corresponding adenoviruses (control adenovirus and adenovirus-delivered shRNA against ALK5) (2.5 × 10^10^ pfu/mL) were injected into the right lateral ventricular of rats (anterior-posterior-1.1 mm, medial-lateral-1.5 mm, dorsal-ventral-4.0 mm from the bregma) (Zhang et al., 2018). Thereafter, MCAO/R modeling was performed using the intraluminal filament method as previously described (Zhang et al., 2018). Specifically, MCAO was performed using the coagulated external carotid artery stump which was sutured by nylon filament and rounded by paraffin wax. After 90 min, blood reperfusion was realized by the removal of filament. Besides, rats with the sham operation were treated similarly except for inserting the suture to the artery. Regional cerebral blood flow (rCBF) and other physiological parameters were observed by a laser-Doppler flowmetry with a body temperature of rats maintained at 37.0 ± 0.5°C using a heating pad. At least a 75% reduction of rCBF should be observed during MCAO/R. Importantly, rats without at least a 75% reduction of rCBF during MCAO or neurological deficits after reperfusion, were excluded from the study. After 14 days of MCAO/R modeling, a 0.2 mL blood sample was collected from the tail vein by liquid chromatography (UltiMate^TM^3000, Thermo Fisher Scientific, Waltham, MA, USA) from a successfully modeled rat followed by the determination of TMAO concentration.

Rats were grouped into control group (control rats), sham group (sham-operated rats), MCAO/R group (MCAO/R rats), MCAO/R + TMAO group (MCAO/R rats injected with TMAO), MCAO/R + TMAO + short hairpin RNA-negative control (sh-NC) group [MCAO/R rats injected with TMAO and sh-NC adenovirus], and MCAO/R + TMAO + sh-ALK5 group (MCAO/R rats injected with TMAO and sh-ALK5 adenovirus) (15 rats for each group). The adenovirus used in this study was purchased from the GeneChem (Shanghai, China) and constructed using the GV119 vector (GeneChem). Primer sequences were also provided by the GeneChem. The experimental procedures were carried out according to the instructions provided by the GeneChem.

### Evaluation of Neurological Function

Fourteen days after the MCAO/R modeling, an evaluation of neurological function was performed by two investigators in a blinded manner. The scoring system comprising seven sub-tests (spontaneous activity, the symmetry of limb movements, forepaw extension, climbing, body proprioception, vibrotactile response, and beam walking). Neurological scores ranged from 3 (the most severe deficit) to 21 (normal).

### Behavioral Evaluation

The staircase test and the cylinder test were performed 14 days after the MCAO/R modeling, while both tests were conducted by an investigator blinded to the experimental conditions. The staircase test was used to evaluate fine motor function. Moreover, before MCAO/R surgery, rats were trained daily for about 1 week. Thereafter, a staircase apparatus with a central platform and seven steps located on either side was prepared. Each stair was prepared with 3 chow pellets (45 mg). The number of pellets grasped by the forelimb of rats was recorded during each 15 min test.

Furthermore, the cylinder test was adopted to assess asymmetries in the forelimb used for postural support. A transparent cylinder (20 cm diameter and 30 cm height) was used in this test. Then, the vertical movement of rats along the cylinder wall was assessed: (a) independent use of the right or left forelimb for contacting the wall, (b) simultaneous use of both the left and right forelimb for contacting the wall, and (c) subsequent use of the other forelimb against the wall following unilateral forelimb placement was scored as “both” as well. Twenty movements were recorded within a 10-min trial with the formula as 100 × (ipsilateral forelimb use + 1/2 bilateral forelimb use)/total forelimb use (Zhang et al., 2018).

### Culture of Primary Astrocytes

Primary astrocytes were isolated from the cerebral cortex of neonatal SD rats (1–3 days). Cerebral cortex was mechanically isolated, detached with 0.25% trypsin (25200056, Gibco, Green Island, NY, USA), centrifuged, and resuspended in a single-cell suspension. The isolated astrocytes were transferred onto a 25 cm^2^ culture flask. Then, Dulbecco's modified Eagle's medium (DMEM)/F-12 (C11330500BT, Gibco) containing 10% fetal bovine serum (FBS; P30-2602, PAN-Biotech, Aidenbach, Germany) and 1% penicillin-streptomycin (15070063, Gibco) was supplemented for incubation at 37°C and 5% CO_2_. The medium was renewed every 3 days. The confluent astrocytes were shaken overnight (at 260 g and 37°C) to remove microglia and oligodendrocytes. Astrocytes attached to the culture flask were trypsinized and sub-cultured. The following experiments were performed when astrocytes were confluent again and more than 95% of the astrocytes were positively reacted to glial fibrillary acidic protein (GFAP). Afterward, astrocytes were infected with lentivirus for 6 h, followed by culturing in normal medium for 2 days and then culturing in medium containing TMAO (100 mmol/L) for 3 days (Gandhi et al., 2010; Zhang et al., 2018).

Astrocytes with no treatment were designated as control while other astrocytes were treated with TMAO, Smurf2 overexpression (oe-Smurf2) lentivirus, oe-NC lentivirus, sh-Smurf2-1 lentivirus, sh-Smurf2-2 lentivirus, sh-NC lentivirus, dimethylsulfoxide (DMSO), MG132, ALK5 overexpression (oe-ALK5) lentivirus, sh-ALK5-1 lentivirus, and sh-ALK5-2 lentivirus alone or in combination. Silencing lentiviruses were packaged with core plasmids (PLKO.1) and accessory plasmids (RRE, REV, Vsvg) inserted with the target gene silencing sequence; core plasmids (Fugw-GFP, Plx304), and accessory plasmids (RRE, REV, Vsvg) inserted with the target gene cDNA sequence were used for the package of overexpressed lentiviruses. All lentiviruses were purchased from Sangon Biotech (Shanghai, China) while primer sequences and plasmid construction were completed by the Sangon Biotech.

### RNA Isolation and Quantification

Total RNA was extracted with the TRIzol reagent (16096020, Thermo Fisher Scientific), and the cDNA of mRNA was synthesized using the first-strand synthesis kit (D7168L, Beyotime Biotechnology Co., Ltd., Shanghai, China) according to the instructions. Reverse transcription-quantitative polymerase chain reaction (RT-qPCR) was carried out using a RT-qPCR kit (Q511-02, Vazyme Biotech, Nanjing, China) according to the instructions. PCR amplification was carried out with Bio-rad real-time quantitative PCR instrument CFX96. β-actin served as the internal reference for Smurf2 and ALK5. All primer sequences were designed and provided by Sangon Biotech. The primer sequences are shown in Table 1. The relative mRNA expression was measured using the 2-^ΔΔ*CT*^ method (Zhao et al., 2018; Wan et al., 2019).

**Table 1:** Primer sequences used for RT-qPCR.

	**Primer sequences**
Smurf2	Forward: 5′-CAGTGGTTTTCCGTGCAGTG-3′
	Reverse: 5′-AGAGCGACTGGAGGAAGGAT-3′
ALK5	Forward: 5′-GCTTCTCATCGTGTTGGTGG-3′
	Reverse: 5′-TGCTTTTCTGTAGTTGGGAGT-3′
β-actin	Forward: 5′-CTGTGTGGATTGGTGGCTCT-3′
	Reverse: 5′-CAGCTCAGTAACAGTCCGCC-3′

### Western Blotting

Total protein was extracted by radioimmunoprecipitation assay (RIPA) lysis buffer containing phenylmethanesulfonyl fluoride (PMSF; P0013B, Beyotime). The nuclear and cytoplasmic proteins were extracted following the instructions of the kit (P0028, Beyotime). The total protein concentration of each sample was then determined by a bicinchoninic acid (BCA) kit (23229, Thermo Fisher Scientific), and the concentration of protein was adjusted to 1 μg/μL. Based on the size of the target protein band, 8%-12% sodium dodecyl sulfate (SDS) gel was prepared, and the protein samples were separated using electrophoresis. Proteins on the gel were transferred onto polyvinylidene fluoride (PVDF) membranes (1620177, Bio-Rad, Hercules, CA, USA). The membranes were blocked with 5% skimmed milk or 5% bovine serum albumin (BSA) for 1 h at room temperature and incubated with diluted primary antibodies against rabbit anti-rat β-actin (ab8227, 1: 5,000, Abcam Inc., Cambridge, UK), mouse anti-rat Smurf2 (ab94483, 1: 1,000, Abcam), rabbit anti-rat ALK5 (ab31013, 1: 1,000, Abcam), rabbit anti-rat GFAP (ab7260, 1: 1,000, Abcam), rabbit anti-rat Neurocan (ab125021, 1: 1,000, Abcam), and rabbit anti-rat Phosphacan (PA5-101832, 1:1,000, Invitrogen Inc., Carlsbad, CA, USA) overnight at 4°C. The next day, the membranes were incubated with horseradish peroxide (HRP)-labeled secondary antibodies of goat anti-rabbit IgG (ab6721, 1: 5,000, Abcam) or rabbit anti-mouse IgG (ab6728, 1: 5,000, Abcam) for 1 h at room temperature. Thereafter, the membrane was immersed in enhanced chemiluminescence (ECL) reaction solution (1705062, Bio-Rad) at room temperature for 1 min. The image was developed on an Image Quant LAS 4000C gel imager (GE Appliances, Orlando, USA). ImageJ2x software was used for the analysis of western blot images. Relative protein expression was measured with β-actin serving as internal reference and expressed as the ratio of gray value of target band to internal reference band (Das et al., 2011).

### 5-ethynyl-2′-Deoxyuridine (EdU) Staining

The cells to be tested were seeded in 24-well plates, and cells in each group were set with 3 duplicated wells. EdU solution (Invitrogen) was added into the culture medium to a concentration of 10 mol/L. Cells were incubated for 2 h followed by the removal of culture medium. Cells were then fixed for 15 min at room temperature with phosphate buffer saline (PBS) solution containing 4% paraformaldehyde, washed twice with PBS containing 3% BSA, and incubated for 20 min at room temperature with PBS containing 0.5% Triton X-100. Then 100 μL of freshly prepared Click-iT staining solution (Invitrogen) was added to each well and incubated for 30 min in the dark at room temperature. Nuclei were stained with 4',6-diamidino-2-phenylindole (DAPI) for 5 min. After mounting, the images were observed under a microscope (BX63, Olympus, Tokyo, Japan) with 6–10 fields randomly chosen. The number of positive cells in each field was recorded following the formula as EdU labeling rate (%) = number of positive cells/(number of positive cells + number of negative cells) × 100% (Ning et al., 2013).

### Cell Counting Kit-8 (CCK-8) Assay

The viability of differently treated cells was assessed following the instructions of the CCK-8 kit (GK10001, GLPBIO, Montclair, CA, USA). Briefly, 10 μL CCK-8 reagent was added to each well and incubated for 1 h. Optical density (OD) values of each well were then detected at a wavelength of 450 nm. The activity of cells in the control group was regarded as 100%, and the activity of cells in the other experimental groups was calculated using the formula: (OD values in the experimental group/OD values in the control group) × 100%. Each experiment was repeated 3 times (Huang et al., 2019).

### Transwell Migration Assay

The migration ability of cells was detected by the Transwell chamber (MCEP24H48, Millipore, Billerica, MA, USA). Astrocytes were cultured under different conditions, trypsinized, and added to the FBS-free upper chamber at a density of 1 × 10^5^ cells. To induce migration, 600 μL FBS-containing medium was added to the basolateral chamber. After 24 h of culture, floating cells and debris were removed from the upper surface of the chamber. Then cells were fixed with cold methanol for 15 min, stained with crystal violet (C0121, Beyotime) for 15 min, and counted under a microscope (BX63, Olympus) in four randomly selected visual fields, with the cell migration rate measured (Yue et al., 2015).

### Immunofluorescence

After rats were anesthetized, the heart was perfused with cold PBS and 4% paraformaldehyde. Brains of rats were separated, fixed, dehydrated, and cut into 10 μm sections. After antigen extraction with sodium citrate buffer, the brain tissue sections were immersed in 0.1% Triton X-100, blocked with 10% normal donkey serum, and incubated with primary rabbit anti-rat antibodies against GFAP (ab7260, 1:200, Abcam), Neurocan (ab31979, 1:300, Abcam), and Phosphacan (PA5-101832, 1:100, Invitrogen) overnight at 4°C. The next day, after PBS washing, the sections were re-probed with Alexa Fluor 555-conjugated goat anti-rabbit immunoglobulin G (IgG) (ab150078, 1:100, Abcam) secondary antibody for 1 h at room temperature. Following after, the sections were sealed with DAPI (C1006, Beyotime) and photographed under a microscope (BX63, Olympus) (Zhang et al., 2018).

### Immunohistochemistry (IHC)

The paraffin sections were baked in an oven at 60°C for 20 min, dewaxed 5 min in each xylene (I, II, III), rehydrated using absolute ethanol (I, II), 90% ethanol (I, II), 80% ethanol (I, II), 70% ethanol (I, II) for 2 min each, washed in water, then transferred to distilled water for 2 min, and washed with PBS for 5 min. For antigen retrieval exposure antigenic determinants: the sections were placed in the Tris-EDTA buffer solution (pH 9.0), heated twice in a microwave oven for 5 min, and blocked for non-specific protein removal. Then the sections were taken out, and the surrounding water was absorbed with filter paper, after which a circle was drawn around the tissue with a histochemical pen, and 5% goat serum was dropped into the circle and put in a humid box at room temperature for 30 min of incubation. Thereafter, the sections were immunostained with primary antibodies against Smurf2 diluted in PBS (sc393848, 1:300, Santa Cruz Biotechnology, Inc., Santa Cruz, CA, USA), rabbit anti-rat ALK5 (ab31013, 1:100, Abcam) overnight at 4°C. The next day, sections were subjected to additional incubation with the diluted secondary antibody goat anti-rabbit (ab6721, 1:500, Abcam, UK) or rabbit anti-mouse (ab6728, 1:500, Abcam) at 37°C for 30 minutes. Following five washes with PBS (5 min each time), the sections were developed by addition of 200 μL 3,3'-diaminobenzidine tetrahydrochloride (DAB) (Shgma, USA) working solution, and incubated for 20 min in the dark, followed by counter-staining, dehydration, clearing, and mounting. After three PBS washes (3 min each time), the sections were stained with approximately 100 μL hematoxylin staining solution for 10 min, blued in PBS for 5 min, dehydrated, cleared, mounted, and observed under a microscope (BX63, Olympus, Japan). The experiment was repeated three times (Lim et al., 2007).

### Co-immunoprecipitation (Co-IP) Assay

The interaction of endogenous Smurf2 with ALK5 protein was detected by the Co-IP. Cells were lysed with Pierce IP buffer [1% Triton X-100, 150 mM NaCl, 1 mM EDTA, 25 mM Tris HCl (pH 7.5)], and protease, followed by the addition of phosphatase inhibitors. Then cell lysate was incubated with mouse anti-rat antibodies to Smurf2 (sc-393848, 1:100, Santa Cruz Biotechnology) and anti-rat IgG (205720, 1:100, Abcam) overnight at 4°C. Cells were then added with protein G-beads (Dynabeads, Thermo Fisher Scientific), rotated slowly at 4°C for 8 h, and finally analyzed by Western blotting (Ambrosi et al., 2016).

### *In vivo* Ubiquitination Assay

For the determination of endogenous ALK5, cells were incubated with 10 μM of ubiquitination inhibitor MG132 (HY-13259, MedChemExpress, Monmouth Junction, NJ, USA) for 6 h, whereas the cells in the control group were added with an equal amount of DMSO (D2650, Sigma-Aldrich). Differently treated cells were lysed in 1% SDS RIPA buffer and subjected to ultrasonication. Subsequently, IP reaction was performed, and diluted cell lysis buffer (the remaining 0.1% SDS) was incubated with ALK5 primary antibody (ab31013, 1:100, Abcam) overnight at 4°C. After being added with protein-G beads and incubated at 4°C for another 8 h, the cells were washed three times in IP buffer. The degree of ALK5 ubiquitination was detected by the western blotting using the rabbit anti-rat Ubiquitin (ab7780, 1:1,000, Abcam) (Chae et al., 2019).

### Statistical Analysis

SPSS 21.0 (IBM Corp., Armonk, NY, USA) software was applied for data statistical analysis. Measurement data were expressed as the mean ± standard deviation, which were derived from three independently repeated experiments. Unpaired *t*-test was adopted for data comparison between two groups, while one-way analysis of variance (ANOVA) was employed for data comparison among multiple groups followed by Tukey's *post hoc* test. Comparison among multiple groups at different time points was performed using repeated-measures ANOVA and Bonferroni's post-test. The difference was statistically significant when *p* < 0.05.

## Results

### TMAO Promotes Proliferation of Reactive Astrocytes and Formation of Glial Scar in MCAO/R Rats

**Figure 1:**
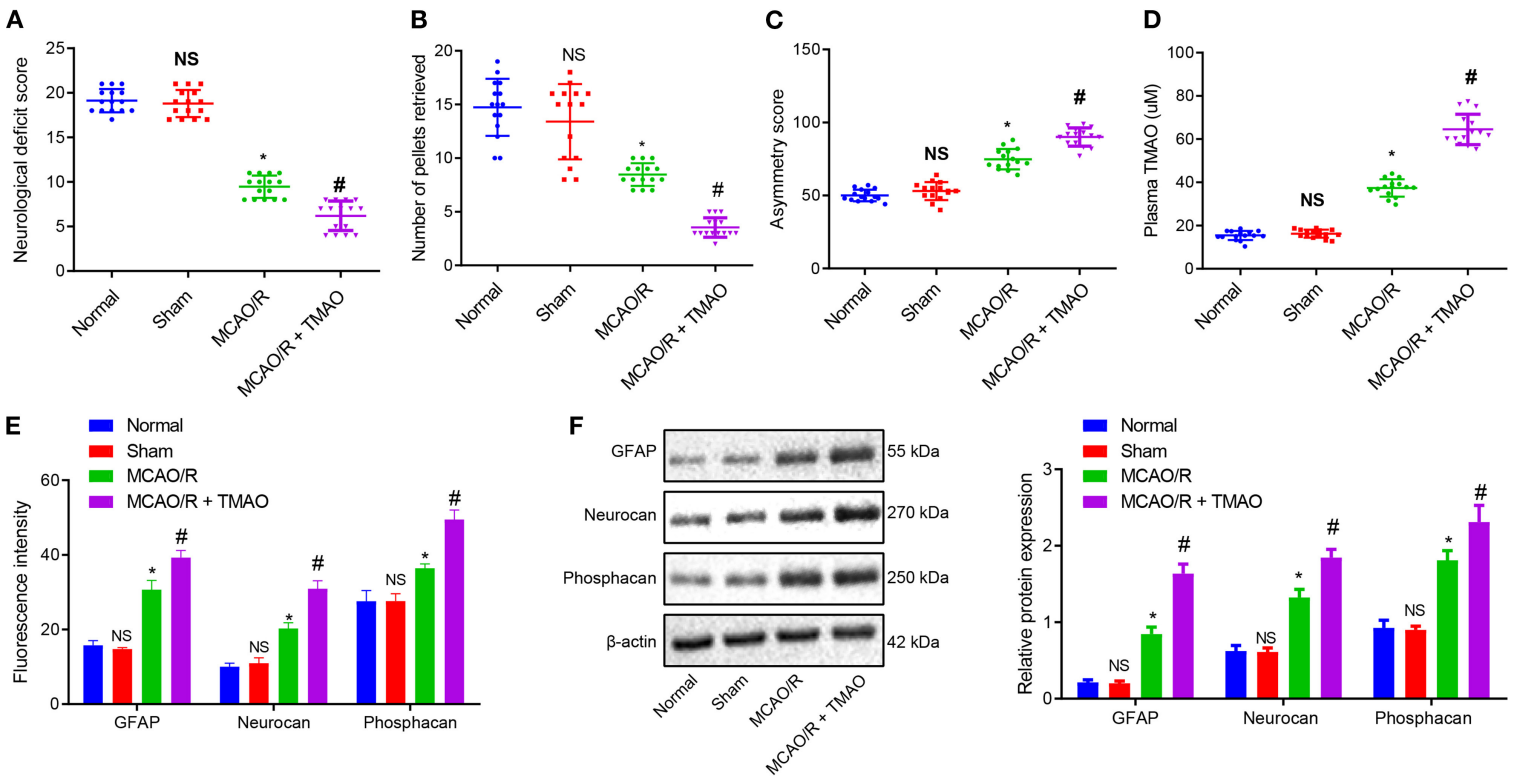
Proliferation of reactive astrocytes and glial scarring is promoted by TMAO. **(A)** Evaluation of neurological function on MCAO/R rats treated with TMAO. **(B)** Number of collected chow pellets detected by staircase test in MCAO/R rats treated with TMAO. **(C)** Scoring results of asymmetry by cylinder test in MCAO/R rats treated with TMAO. **(D)** Statistical results of TMAO concentration in brain tissues of MCAO/R rats treated with TMAO. **(E)** Expression of GFAP, Neurocan, and Phophacan detected by immunofluorescence assay in brain tissues of MCAO/R rats treated with TMAO. **(F)** Expression of GFAP, Neurocan, and Phosphacan determined by Western blotting in brain tissues of MCAO/R rats treated with TMAO. *p < 0.05 compared with sham-operated rats (*n* = 15). #*p* < 0.05 compared with MCAO/R rats (*n* = 15). NS *p* > 0.05 compared with the control rats (*n* = 15). One-way ANOVA is employed for data comparison among multiple groups followed by Tukey's *post hoc* test.

To verify the effect of TMAO on ischemic stroke, an MCAO/R rat model was constructed, followed by the identification of the MCAO/R rat modeling. The results of neurological function scoring showed that compared with sham-operated rats, the neurological function score of MCAO/R rats was decreased. Compared with MCAO/R rats, the neurological function score of MCAO/R rats treated with TMAO was distinctly reduced (Figure 1A). The behavioral evaluation results showed that the number of chow pellets collected by MCAO/R rats was decreased whereas the asymmetric score was significantly increased. Intriguingly, similar results were observed in the MCAO/R rats treated with TMAO when compared to MCAO/R rats (Figures 1B,C). These results indicated that neurological recovery was poor in MCAO/R rats and much worsen in MCAO/R rats treated with TMAO. Moreover, the concentration of TMAO measured by a liquid phase spectrometer was elevated in the MCAO/R rats when compared with sham-operated rats. In contrast to MCAO/R rats, the concentration of TMAO was significantly increased in MCAO/R rats treated with TMAO (Figure 1D).

To further evaluate the effects of TMAO on the proliferation of reactive astrocytes and glial scar formation in MCAO/R rats, the expression of GFAP, Neurocan, and Phosphacan was detected in the glial scar by immunofluorescence and Western blotting. The results showed that the expression of GFAP, Neurocan, and Phosphacan in brain tissues of MCAO/R rats was remarkably increased, and this increase was further promoted by treatment with TMAO (Figures 1E,F). Collectively, these results suggest the possible ability of TMAO to potentiate the reactive astrocyte proliferation and glial scar formation in MCAO/R rats.

### Overexpression of Smurf2 Inhibits the TMAO-Induced Astrocyte Proliferation, Migration, and Activation

**Figure 2:**
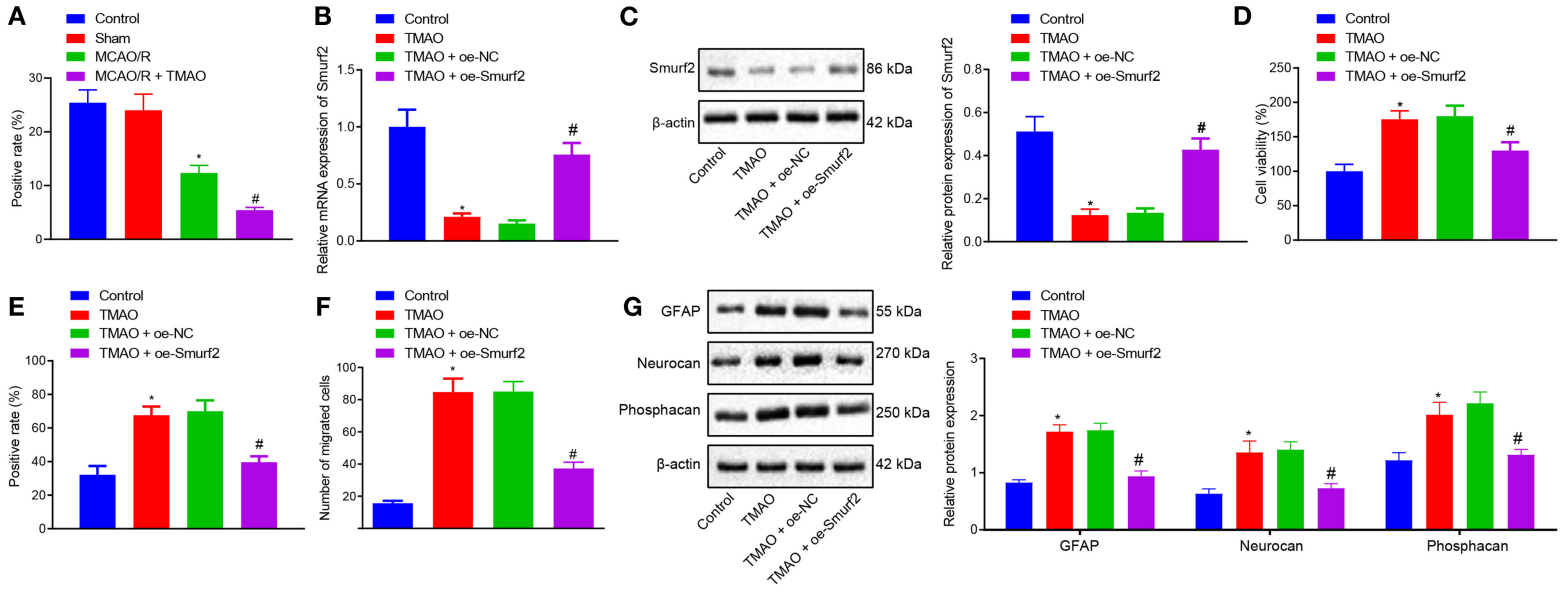
Overexpression of Smurf2 suppresses the promoting effect of TMAO on proliferation, migration, and viability of astrocytes. **(A)** Smurf2 expression in rat brain tissues (*n* = 15) detected by IHC assay. **(B)** Smurf2 expression in differently treated cells determined by RT-qPCR. **(C)** Smurf2 expression determined by western blotting in differently treated cells. **(D)** Results of CCK-8 assay on cell viability upon different treatments. **(E)** Proliferation of cells upon different treatments detected by EdU assay. **(F)** Migration ability of cells upon different treatments assessed by Transwell migration assay. **(G)** Protein expression of GFAP, Neurocan and Phosphacan determined using Western blotting in differently treated cells. *p < 0.05 compared with the control group. #*p* < 0.05 compared with the TMAO + oe-NC group. One-way ANOVA was employed for data comparison among multiple groups followed by Tukey's *post hoc* test.

To verify our assumption that TMAO might promote reactive astrocyte proliferation and glial scar formation by inhibiting the Smurf2 expression, Smurf2 expression in glial scar was assessed by the IHC. Interestingly, our results showed that Smurf2 expression was significantly reduced in brain tissues of MCAO/R rats than in sham-operated rats, and a reduction was also observed in brain tissues of MCAO/R rats administrated with TMAO compared with MCAO/R rats (Figure 2A; Supplementary Figure 1A). Additionally, the results of RT-qPCR and Western blotting showed a significant decline of Smurf2 expression in TMAO-treated astrocytes (*p* < 0.05), whereas, a significant increase was noted in the presence of TMAO + oe-Smurf2 (*p* < 0.05; Figures 2B,C).

The viability of differently treated cells was evaluated using CCK-8 assay, the results of which showed that TMAO-treated astrocytes exhibited enhanced cell viability while overexpression of Smurf2 curtailed cell viability in TMAO-treated astrocytes (Figure 2D). Besides, EdU and Transwell assay results displayed that the proliferation and migration ability of TMAO-treated astrocytes was distinctly increased, yet, TMAO-treated astrocytes (overexpressing Smurf2) were found to be dampened proliferation and migration (Figures 2E,F; Supplementary Figures 1B,C). Furthermore, the expression of GFAP, Neurocan, and Phosphacan determined by Western blotting showed an upward trend in the TMAO-treated astrocytes, while the opposite results were observed following overexpression of Smurf2 (Figure 2G). Taken together, the above data exhibited that overexpression of Smurf2 can abolish the promotion of astrocyte proliferation, migration, and viability induced by TMAO.

### TMAO Promotes ALK5 Expression by Inhibiting the Ubiquitination Function of Smurf2

**Figure 3:**
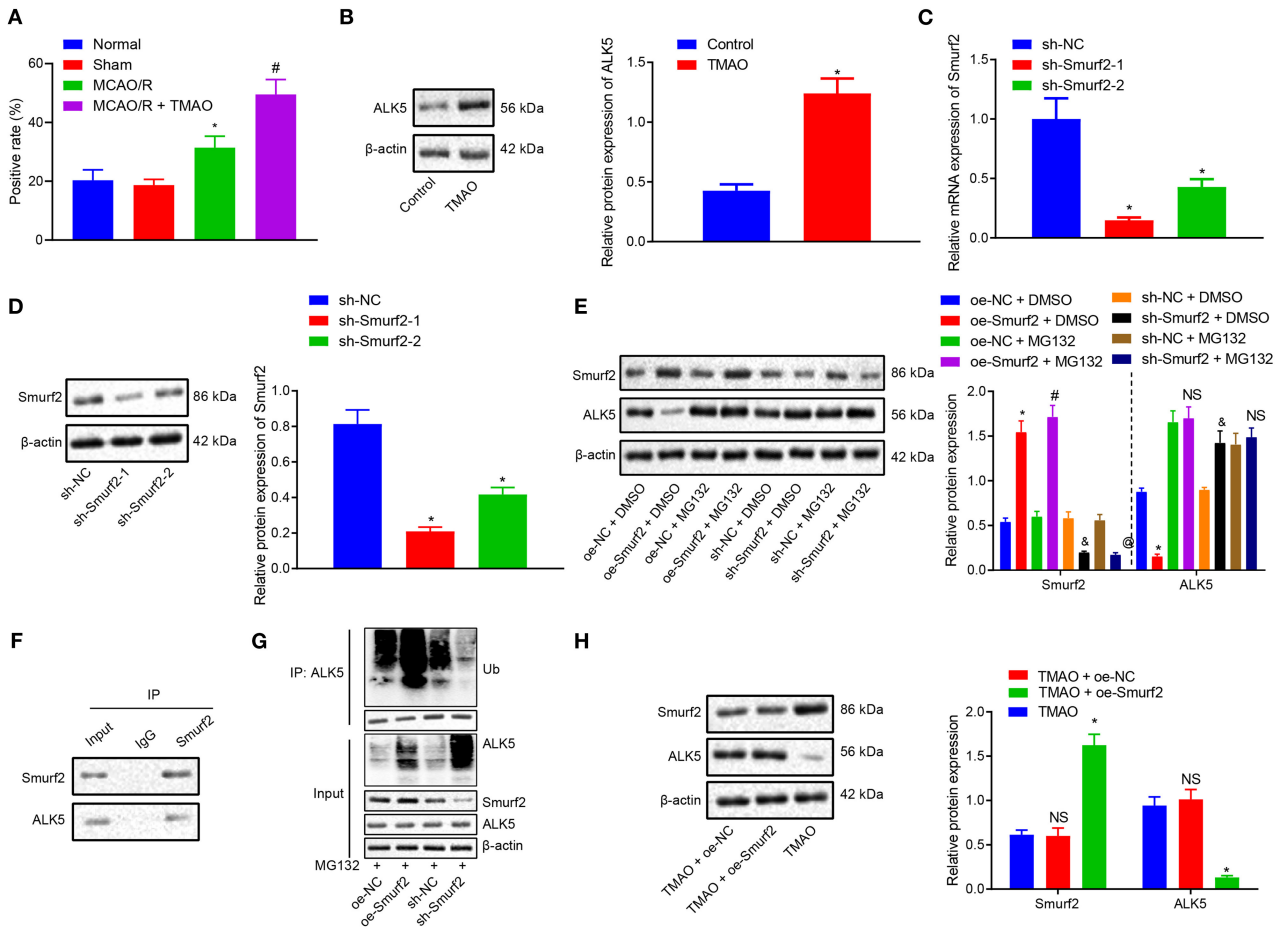
TMAO promotes the expression of ALK5 *via* disruption of the Smuf2 ubiquitination function. **(A)** IHC results of ALK5 expression. **(B)** ALK5 expression determined by Western blotting in astrocytes. **(C)** Smurf2 expression determined by RT-qPCR in astrocytes. **(D)** Smurf2 expression determined by Western blotting in astrocytes. **(E)** ALK5 protein expression detected by Western blotting. **(F)** IP assay to detect the interaction between Smurf2 and ALK5 in astrocytes. **(G)** IP assay to detect the degree of ALK5 ubiquitination. **(H)** Protein expression of ALK5 in astrocytes assessed by Western blotting. *p < 0.05 compared with the control, sh-NC, oe-NC + DMSO or TMAO + oe-NC group. #*p* < 0.05 compared with the oe-NC + MG132 group. & *p* < 0.05 compared with the sh-NC + DMSO group. @ *p* < 0.05 compared with the sh-NC + MG132 group. NS indicates the comparison is not statistically significant. Unpaired *t*-test is adopted for data comparison between two groups. One-way ANOVA is employed for data comparison among multiple groups followed by Tukey's *post hoc* test.

To further investigate the downstream mechanism underlying TMAO/Smurf2, expression of ALK5 in brain tissues of sham-operated rats, MCAO/R rats, and MCAO/R rats treated with TMAO was detected by IHC. Our results showed that ALK5 expression was elevated in brain tissues of MCAO/R rats, which was also promoted in brain tissues of MCAO/R rats treated with TMAO (Figure 3A; Supplementary Figure 2). Moreover, the expression of ALK5 in astrocytes was determined by the western blot analysis, the results of which showed an enhancement in astrocytes treated with TMAO in comparison to control astrocytes (Figure 3B). Thereafter, the silencing efficiency of Smurf2 in astrocytes was detected by RT-qPCR and western blotting. The results showed that compared with astrocytes treated with sh-NC, the expression of Smurf2 was significantly decreased in astrocytes treated with sh-Smurf2-1 or sh-Smurf2-2 (Figures 3C,D). Since the silencing efficiency of sh-Smurf2-1 was higher than that of sh-Smurf2-2, subsequent experiments were conducted with sh-Smurf2-1 to knockdown the expression of Smurf2. Besides, the efficiency of Smurf2 overexpression was also verified and the results showed that oe-Smurf2 could successfully increase the expression of Smurf2 (Figure 2B).

Nonetheless, our results of western blotting showed that the protein expression of ALK5 was decreased in astrocytes treated with oe-Smurf2 while it was significantly increased in astrocytes treated with sh-Smurf2. However, upon addition of MG132, the protein expression of ALK5 did not significantly change (Figure 3E), suggesting the correlation of the Smurf2 ubiquitination with the ALK5 expression. To further explore whether Smurf2 affects the expression of ALK5, we used Co-IP assay to verify the interaction between Smurf2 and ALK5 in cells and the results showed the interaction of Smurf2 with ALK5 at the endogenous level (Figure 3F). Moreover, the endogenous level of ubiquitination was detected by ubiquitination assay, which showed that the degree of ALK5 ubiquitination was enhanced in the astrocytes overexpressing Smurf2, whilst it was reduced in the astrocytes silencing Smurf2 (Figure 3G). These results suggested that Smurf2 enhanced the ubiquitination and degradation of ALK5. Furthermore, the results of Western blotting showed that the expression of Smurf2 was upregulated while that of ALK5 was downregulated in astrocytes overexpressing both TMAO and Smurf2 (Figure 3H). Hence, the aforementioned data suggested that TMAO could potentially promote ALK5 expression by suppressing the ubiquitination function of Smurf2.

### Overexpression of ALK5 Reverses the Inhibitory Effects of Smurf2 on Astrocyte Proliferation, Migration, and Viability

**Figure 4:**
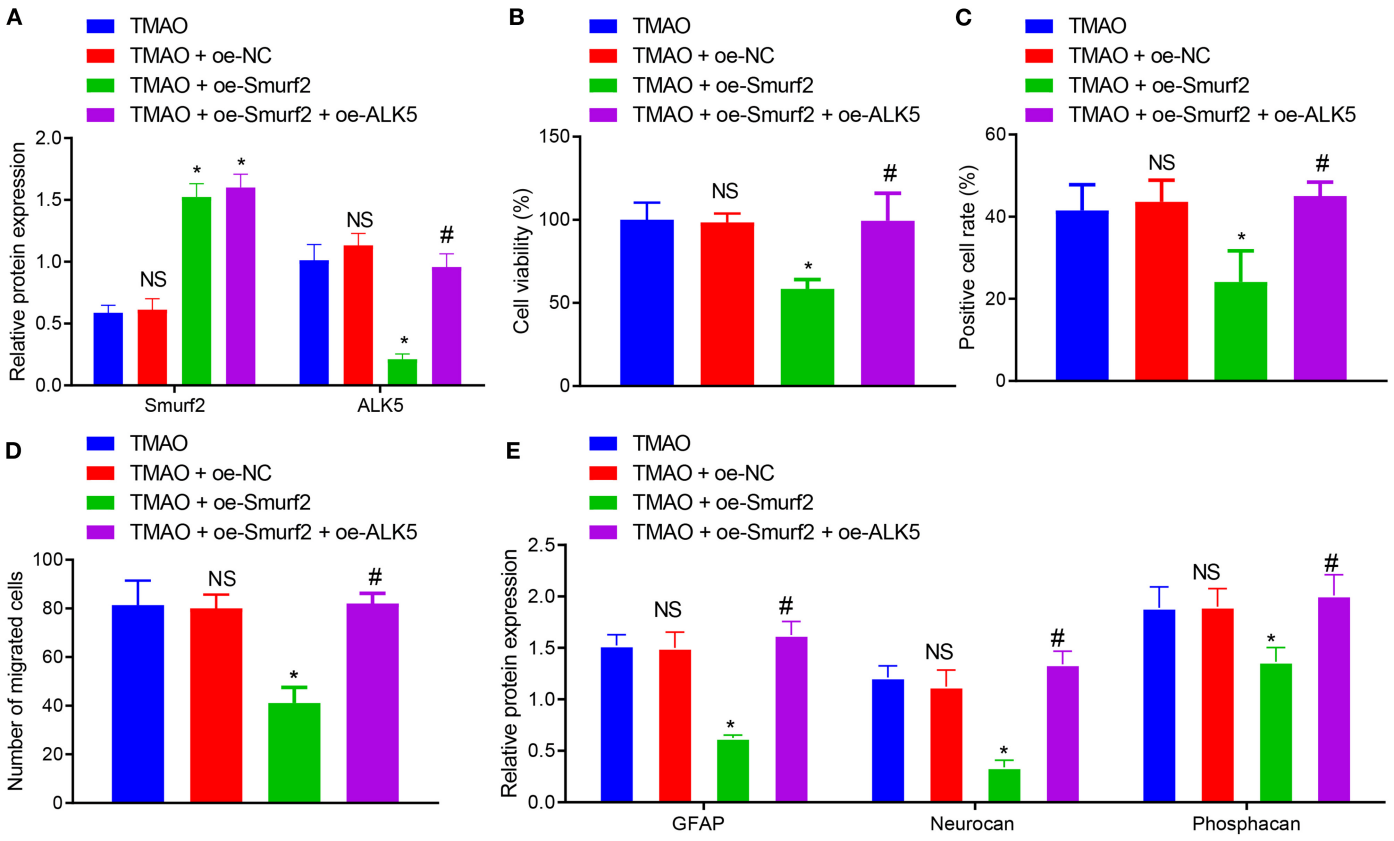
Overexpression of ALK5 disrupts Smurf2-induced inhibition of astrocyte proliferation, migration and viability. **(A)** Expression of Smurf2 and ALK5 determined by Western blotting in each group of cells. **(B)** CCK-8 assay results of cell viability in each group. **(C)** EdU assay results of cell proliferation ability of each group. **(D)** Migration ability measured by Transwell migration assay in cells. **(E)** The expression of GFAP, Neurocan and Phosphacan in each group of cells detected by Western blotting. *p < 0.05 compared with the TMAO + oe-NC group. #*p* < 0.05 compared with the TMAO + oe-Smurf2 group. NS, *p* < 0.05 compared with the TMAO group. One-way ANOVA is employed for data comparison among multiple groups followed by Tukey's *post hoc* test.

To further explore the degradation of ALK5 by ubiquitination of Smurf2 to regulate astrocyte proliferation, migration, and activation, the expression of Smurf2 and ALK5 in differently treated astrocytes was determined by the western blotting. The results showed that the expression of Smurf2 was increased in TMAO-treated astrocytes overexpressing Smurf2 while ALK5 expression was distinctly reduced. Moreover, in TMAO-treated cells overexpressing both Smurf2 and ALK5, Smurf2 expression did not exhibit any significant change while ALK5 expression was enhanced (Figure 4A).

Based on the results of CCK-8, EdU, and Transwell assays, the cell viability, proliferation, and migration were curtailed in TMAO-treated cells overexpressing Smurf2, which was neutralized by overexpression of ALK5 (Figures 4B–D). Additionally, western blot assay revealed that the expression of GFAP, Neurocan, and Phosphacan in astrocytes co-treated with TMAO and oe-Smurf2 was reduced, which was normalized following overexpression of ALK5 (Figure 4E). These results indicated that overexpression of ALK5 may neutralize the inhibitory effect of Smurf2 on astrocyte proliferation, migration, and viability.

### Silencing of ALK5 Reverses the Promoting Effect of TMAO on Astrocyte Proliferation, Migration, and Activation

**Figure 5:**
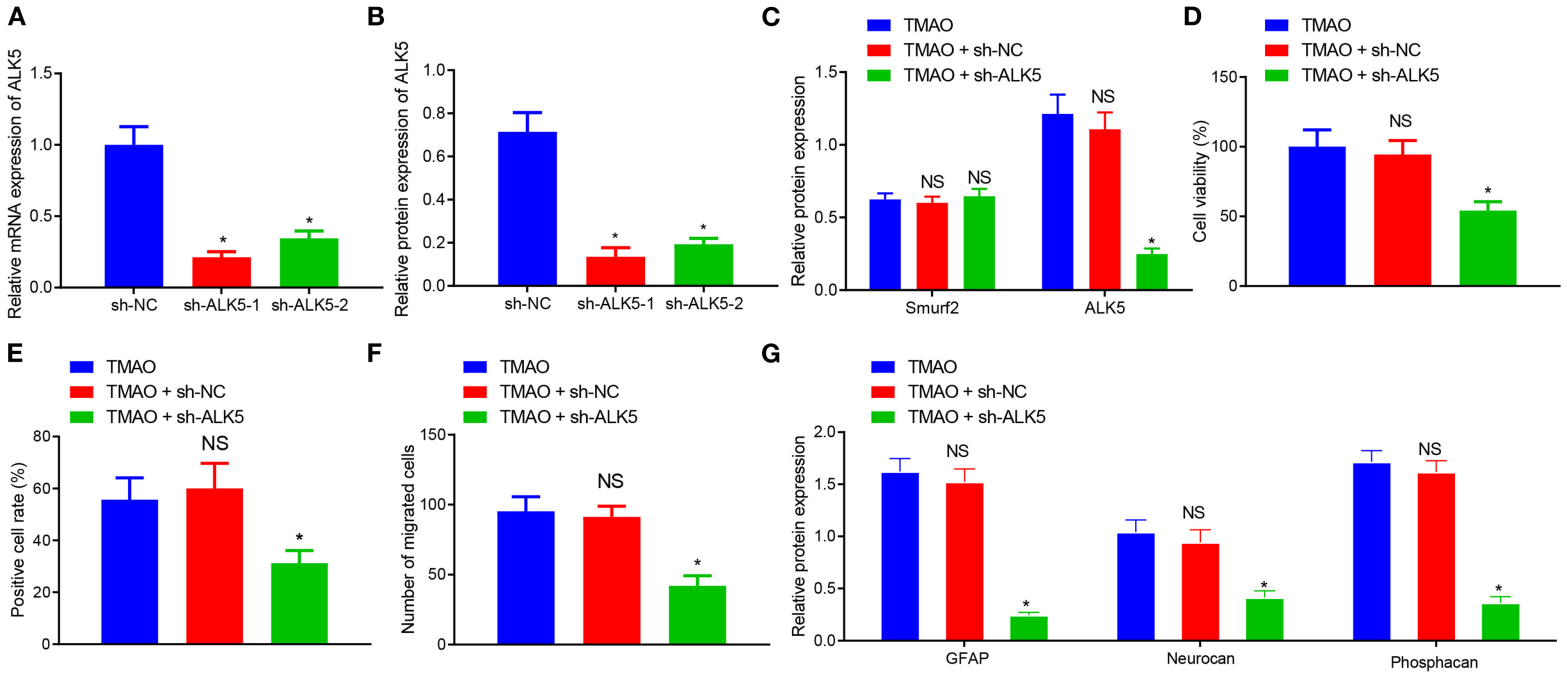
Silencing of ALK5 attenuates the promoting effect of TMAO on astrocyte proliferation, migration and viability. **(A)** Transfection efficiency of ALK5 in cells examined using RT-qPCR. **(B)** Protein expression of ALK5 in cells analyzed using Western blotting. **(C)** Expression of ALK5 and Smurf2 in cells determined by Western blotting. **(D)** Cell viability evaluated using CCK-8 assay. **(E)** Cell proliferation evaluated using EdU assay. **(F)**, Cell migration evaluated using Transwell assay. **(G)**, The expression of GFAP, Neurocan and Phosphacan in each group of cells detected by Western blotting. *p < 0.05 compared with the sh-NC group or the TMAO + oe-NC group. NS, *p* > 0.05 compared with the TMAO group. Unpaired *t*-test is adopted for data comparison between two groups. One-way ANOVA is employed for data comparison among multiple groups followed by Tukey's *post hoc* test.

To further validate the effects of silenced ALK5 on astrocyte proliferation and glial scar formation, ALK5 expression was silenced by the transfection of plasmids in TMAO-treated astrocytes, followed by the detection of transfection efficiency on ALK5 by RT-qPCR and western blot assay. The results showed that the expression of ALK5 was prominently reduced in astrocytes treated with sh-ALK5-1 and sh-ALK5-2 (Figures 5A,B). Since the silencing efficiency of sh-ALK5-1 was higher than that of sh-ALK5-2, subsequent experiments were carried out with sh-ALK5-1 for ALK5 silencing. Western blotting results further demonstrated that the expression of Smurf2 did not exhibit any variation while ALK5 expression was downregulated in astrocytes co-treated with TMAO and sh-ALK5 (Figure 5C).

The results obtained from CCK-8, EdU, and Transwell assays displayed that the cell viability, proliferation, and migration were reduced in cells co-treated with TMAO and sh-ALK5 (Figures 5D–F). The results of Western blotting further exhibited that the expression of GFAP, Neurocan, and Phosphacan was downregulated in TMAO-treated astrocytes silencing ALK5 (Figure 5G). Collectively, these results indicated that the silencing of ALK5 can inhibit the promoting effect of TMAO on astrocyte proliferation, migration, and viability.

### TMAO Promotes the Proliferation of Reactive Astrocytes and Glial Scarring Through the Smurf2/ALK5 Axis

**Figure 6:**
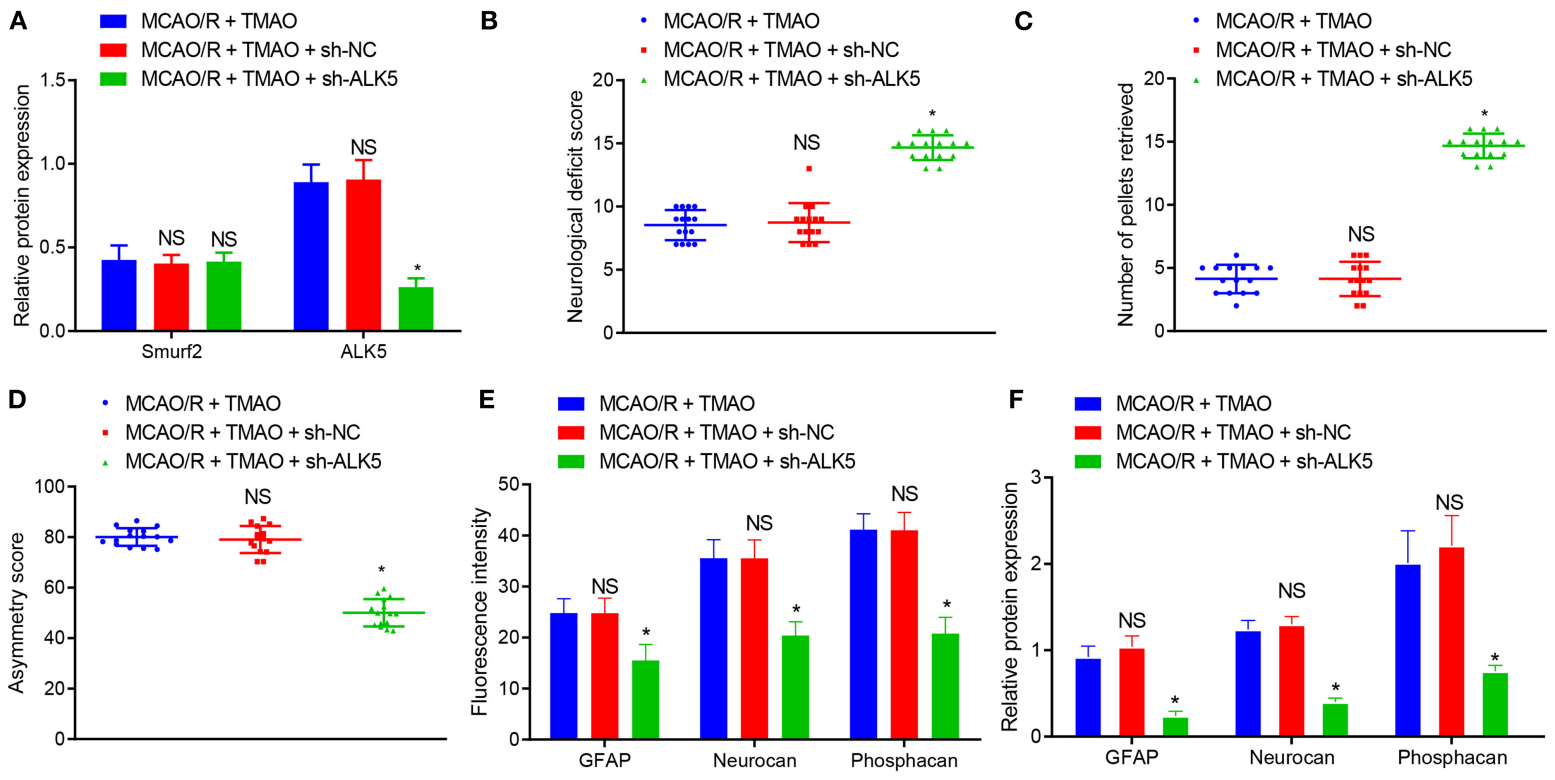
TMAO promotes reactive astrocyte proliferation and glial scarring through the Smurf2/ALK5 axis. **(A)** Smurf2 and ALK5 expression determined by western blotting in brain tissues of MCAO/R rats. **(B)** Neurological scoring of rats after different treatments. **(C)** The number of collected chow pellets by rats in each group detected by staircase test. **(D)** Asymmetric function detected by cylinder test. **(E)** Expression of GFAP, Neurocan and Phosphacan detected by immunofluorescence assay in brain tissues of rats after different treatments. **(F)** Protein expression of GFAP, Neurocan and Phosphacan determined using Western blotting in brain tissues of rats after different treatments. *p < 0.05 compared with MCAO/R + TMAO + sh-NC group (*n* = 15). NS *p* > 0.05 compared with MCAO/R + TMAO group (*n* = 15). One-way ANOVA is employed for data comparison among multiple groups followed by Tukey's *post hoc* test.

Thereafter, we further attempted to validate the effects of TMAO, Smurf2, and ALK5 on reactive astrocyte proliferation and glial scar formation in rats. For this purpose, MCAO/R rats were injected with relevant adenoviruses to increase/decrease expression of TMAO, Smurf2 or ALK5, followed by the determination of the expression of Smurf2 and ALK5 by western blotting. The results exhibited that Smurf2 expression was not significantly different and the expression of ALK5 was decreased in brain tissues of MCAO/R rats co-treated with TMAO and sh-ALK5 (Figure 6A). Meanwhile, the results of neurological function scoring showed that the neurological function score was significantly increased in MCAO/R rats co-treated with TMAO and sh-ALK5 (Figure 6B). Behavioral evaluation results showed that the number of chow pellets collected on day 14 by MCAO/R rats co-treated with TMAO and sh-ALK5 was significantly increased whereas the asymmetric score was reduced (Figures 6C,D). The results of both immunofluorescence and western blotting exhibited that the expression of GFAP, Neurocan, and Phosphacan was downregulated in brain tissues of MCAO/R rats co-treated with TMAO and sh-ALK5 (Figures 6E,F). These results suggest the potential of TMAO to promote reactive astrocyte proliferation and glial scarring through the Smurf2/ALK5 axis.

## Discussion

Recently, accumulating research has brought attention to the therapeutic implication of TMAO, especially in cardiovascular diseases to manage hydrostatic and osmotic stress (Janeiro et al., 2018; Ufnal and Nowinski, 2019). However, the comprehensive underlying mechanism remained unknown. Thus, in the present study, we attempted to explore the functional role of TMAO specific for the restoration of neurological function following ischemic stroke in rats. Collectively, our experimental data demonstrated that TMAO promoted reactive astrogliosis and glial scar formation to curb the neurological function restoration by upregulating the ALK5 through Smurf2 inhibition.

Accordingly, a recently reported study has indicated the increased level of TMAO during the acute stage following the onset of ischemic stroke in a time-dependent manner (Schneider et al., 2020). Moreover, a remarkably increased level of TMAO in the plasma has been detected in ischemic stroke-atrial fibrillation patients when compared with patients with atrial fibrillation (Liang et al., 2019). Consistently, our study reported the high level of TMAO in rats induced with MACO/R in association with poor recovery of neurological function. Moreover, consistent with our data, a previous study has also reported the elevated level of TMAO in the plasma or serum of patients which foreshadows the higher risks of dismal functional outcome and mortality (Rexidamu et al., 2019; Zhai et al., 2019). Besides, TMAO treatment has been found to impair the memory and learning abilities of aging mice and enhance the astrocyte activation in astrocytes (Brunt et al., 2020). To further facilitate the recovery of neurological function, reactive astrogliosis has been demonstrated to play a vital role in promoting neuronal plasticity (Sims and Yew, 2017). Based on the findings of our study, TMAO treatment was observed to enhance the reactive astrogliosis and glial scar formation accompanied by an elevated level of GFAP, Neurocan, and Phosphacan. In addition to reactive astrogliosis, the formation of the glial scar has been indicated to suppress axonal regeneration during the recovery characterized by increased expression of GFAP, Neurocan, and Phosphacan, while sevoflurane post-conditioning has been shown with neuroprotective action by reversing these results (Zhu et al., 2017). Hereby, subsequent attempts were made to explore the potential way to suppress the promoting role of TMAO in ischemic stroke.

Notably, Smurf2 has been reported to be intensively associated with the diagnosis of atrial fibrillation and stroke, which was identified from a protein-protein interaction (PPI) network previously constructed (Zhang et al., 2020). Additionally, Smurf2 can enhance neuron differentiation and improve functional recovery from ischemic stroke by ubiquitinating and degrading the EZH2 (Yu et al., 2013). TMAO has been elucidated to exacerbate cardiac function and fibrosis by downregulating Smurf2 (Yang et al., 2019). Accordingly, a series of gain- and loss-of-function assays in our study underscored the inhibitory action of overexpressed Smurf2 on auxo-action of TMAO on astrogliosis, migration, and activation, which was indicated by diminished expression of GFAP, Neurocan, and Phosphacan. Peculiarly, GFAP has been defined as a complementary biomarker for the prediction of functional outcome in patients suffering from acute ischemic stroke (Liu and Geng, 2018). However, reduced Neurocan level in reactive astrocytes has been demonstrated to enhance the axonal regeneration, thus playing a catalytic role in the neurorestorative action of bone marrow stromal cells (Shen et al., 2008). Likewise, decreased Phosphacan expression has been observed in MCAO-treated mice when the robust expression of early growth response-1 in reactive astrocytes was deficient (Beck et al., 2008). In consent with our results, glial scar markers like GFAP, Neurocan, and Phosphacan, have been demonstrated to be downregulated by the administration of CID1067700 (a Rab7 receptor antagonist involved in the formation of astral sclerosis and glial scars caused by ischemic stroke), which was attributed to the suppressed astrogliosis and glial scar formation as well as improved neurologic deficits as observed in a rat model of ischemic stroke (Qin et al., 2019).

Furthermore, our data from the mechanistic investigation revealed that Smurf2 enhanced the ubiquitination and degradation of ALK5. Similarly, the increased ubiquitination and degradation of specific mRNAs can contribute to their decreased expression (Santibanez et al., 2007; Yang et al., 2020). Smurf2 and ALK5 (TGFβ receptor type I) have been reported as downstream proteins of the TGFβ signaling system in vascular smooth muscle cells (Pan et al., 2007). In consent with our findings, the regulatory role of Smurf2 has been reported to promote ubiquitination and degradation of ALK5 (Chae et al., 2019). On the other hand, TMAO has been reported to increase the ALK5 expression, whereas the ubiquitination of ALK5 has been reported to be blocked by the TMAO treatment in neonatal mouse fibroblasts (Yang et al., 2019). Intriguingly, these above-mentioned findings are in agreement with our present results, indicating that TMAO could increase the ALK5 expression by inhibiting the ubiquitination role of Smurf2. Importantly, TGFβ1 has been documented to stimulate the expression of repulsive guidance molecule to curb the restoration of neurological function by promoting reactive astrogliosis and glial scar formation (Zhang et al., 2018). Besides, it is also noteworthy that the overexpression of ALK5 (a major causal factor of neurogenesis in adults) has been demonstrated to promote neurogenesis and functional recovery (Zhang et al., 2019), which is contrary to our results that silencing of ALK5 repressed the astrogliosis, migration, and activation induced by TMAO in contribution to the recovery of neurological function. This suggests the need for further investigations to validate the results of the present study to identify the activators of the above-mentioned differences.

**Figure 7:**
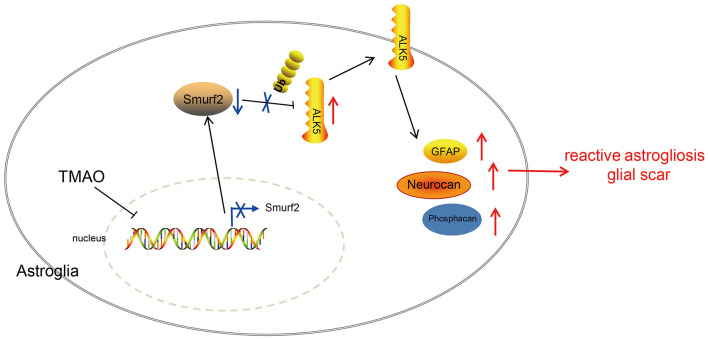
Graphic presentation of molecular mechanism underlying TMAO in ischemic stroke. TMAO promoted the expression of ALK5 by downregulating Smurf2, thereby inhibiting the recovery of neurological function in ischemic stroke and promoting reactive astrocyte proliferation and glial scar formation.

In summary, the results in the present study underscored that TMAO contributed to reactive astrogliosis and glial scar formation during ischemic stroke, leading to suppressed neurological function restoration. Furthermore, the functional role of TMAO was found to depend on the downregulation of Smurf2 and upregulation of ALK5 (Figure 7). This pathway appears to be clinically important for the development of novel therapeutic targets for the alleviation of ischemic stroke. However, at this stage, we cannot exclude the possible involvement of other signaling pathways in the neuroprotection of the TMAO/Smurf2/ALK5 axis due to the complex microenvironments. Additionally, further investigation is required to detect the physiological and pathophysiological differences to correlate the animal results to the human clinical setting. Hence, we recommend a further experimental study on humans to determine the clinical application value of the TMAO/Smurf2/ALK5 axis in ischemic stroke.

## Data Availability Statement

The original contributions presented in the study are included in the article/Supplementary Material, further inquiries can be directed to the corresponding author/s.

## Ethics Statement

The animal study was reviewed and approved by the Animal Ethics Committee of Peking University Shenzhen Hospital.

## Author Contributions

HS designed the study. SF and HQ collated the data, carried out data analyses, and produced the initial draft of the manuscript. LZ contributed to drafting the manuscript. All authors have read and approved the final submitted manuscript.

## Conflict of Interest

The authors declare that the research was conducted in the absence of any commercial or financial relationships that could be construed as a potential conflict of interest.

## Supplementary Material

Supplementary Figure 1 Representative images. **(A)** Representative images of Smurf2 expression in rat brain tissues detected by IHC assay. **(B)** Representative images of proliferation of cells upon different treatments detected by EdU assay. **(C)** Representative images of migration ability of cells upon different treatments assessed by Transwell migration assay.

Supplementary Figure 2 Representative images of IHC results of ALK5 expression.
