# Supplementary figures and images for: TMAO Aggregates Neurological Damage Following Ischemic Stroke by Promoting Reactive Astrocytosis and Glial Scar Formation via the Smurf2/ALK5 Axis
Source: Front Cell Neurosci. 2021 Mar 18;15:569424. doi: 10.3389/fncel.2021.569424 (PMC8012716; doi:10.3389/fncel.2021.569424)

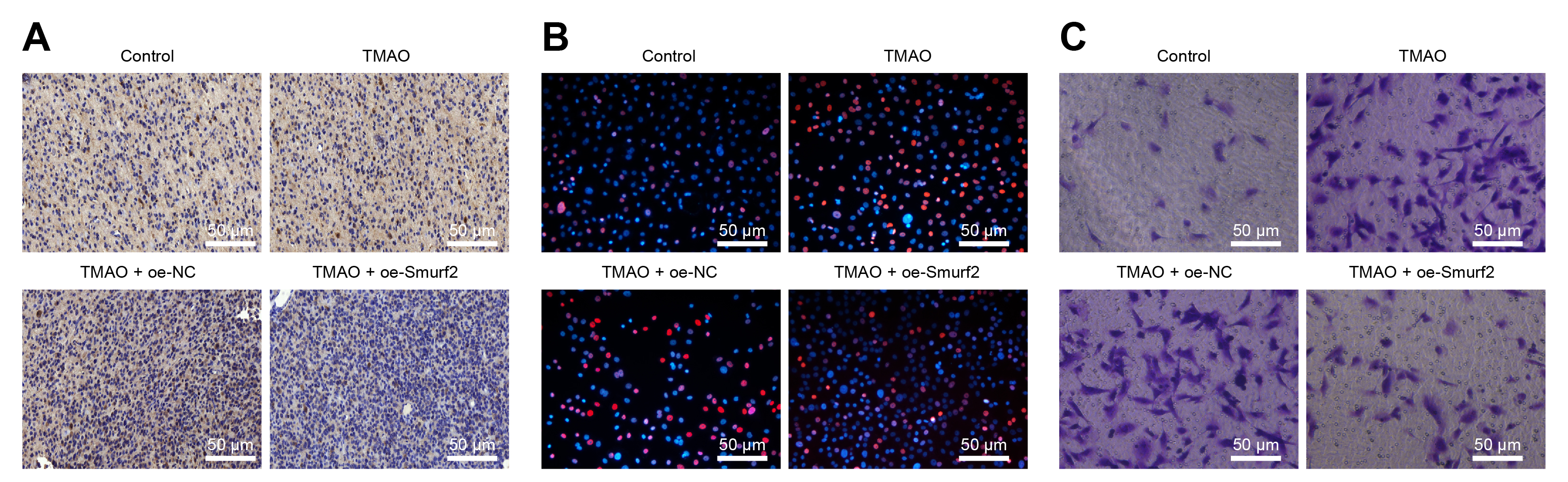

Supplement: Supplementary Figure 1 — Representative images. (A) Representative images of Smurf2 expression in rat brain tissues detected by IHC assay. (B) Representative images of proliferation of cells upon different treatments detected by EdU assay. (C) Representative images of migration ability of cells upon different treatments assessed by Transwell migration assay. [file Image_1.JPEG]

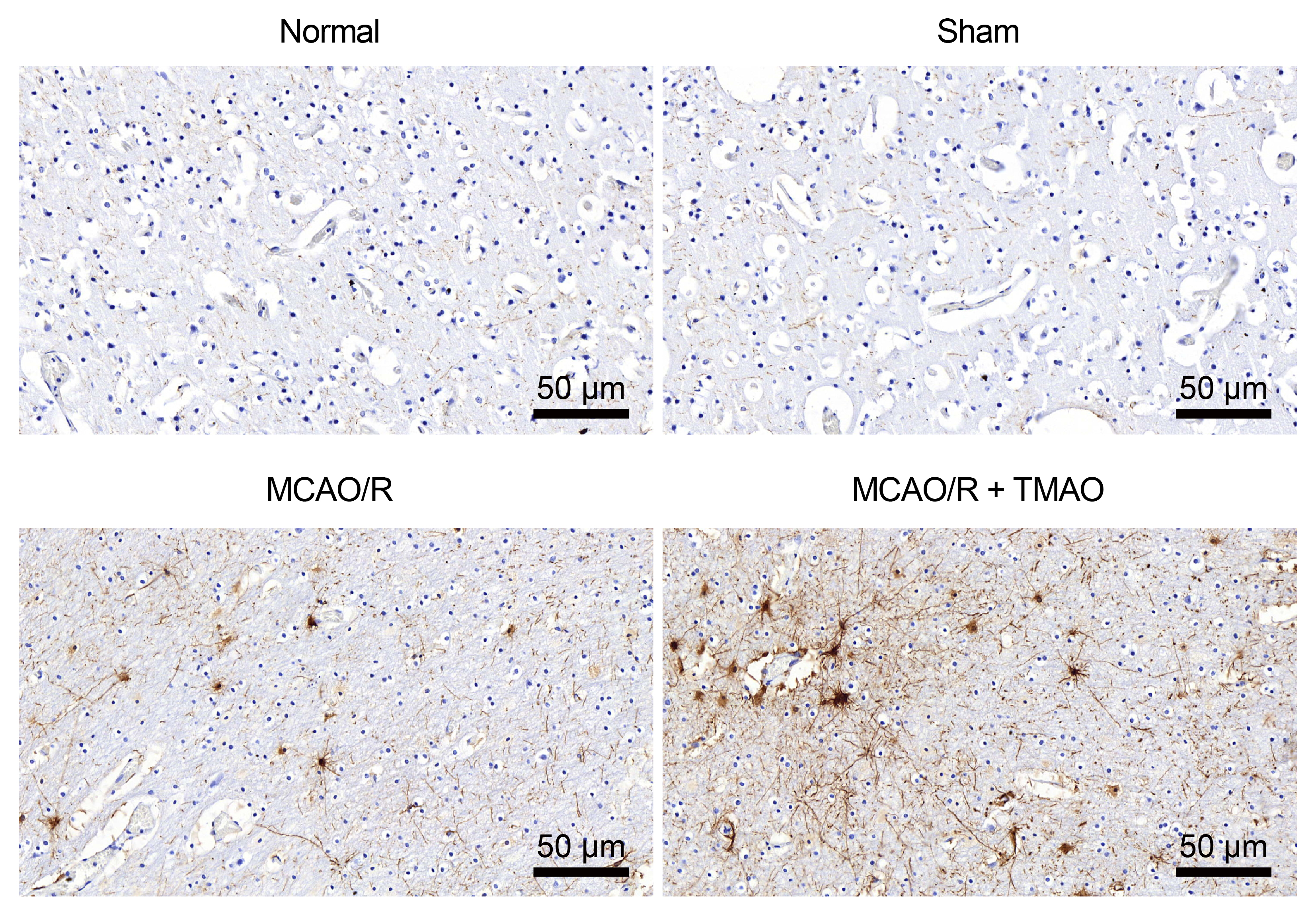

Supplement: Supplementary Figure 2 — Representative images of IHC results of ALK5 expression. [file Image_2.JPEG]
